# Patterns of Early Gut Colonization Shape Future Immune Responses of the Host

**DOI:** 10.1371/journal.pone.0034043

**Published:** 2012-03-27

**Authors:** Camilla Hartmann Friis Hansen, Dennis Sandris Nielsen, Miloslav Kverka, Zuzana Zakostelska, Klara Klimesova, Tomas Hudcovic, Helena Tlaskalova-Hogenova, Axel Kornerup Hansen

**Affiliations:** 1 Department of Veterinary Disease Biology, Faculty of Life Sciences, University of Copenhagen, Frederiksberg C, Denmark; 2 Department of Food Science, Faculty of Life Sciences, University of Copenhagen, Frederiksberg C, Denmark; 3 Department of Immunology and Gnotobiology, Institute of Microbiology, Academy of Sciences of the Czech Republic, Prague and Novy Hradek, Czech Republic; University of Cape Town, South Africa

## Abstract

The most important trigger for immune system development is the exposure to microbial components immediately after birth. Moreover, targeted manipulation of the microbiota can be used to change host susceptibility to immune-mediated diseases. Our aim was to analyze how differences in early gut colonization patterns change the composition of the resident microbiota and future immune system reactivity. Germ-free (GF) mice were either inoculated by single oral gavage of caecal content or let colonized by co-housing with specific pathogen-free (SPF) mice at different time points in the postnatal period. The microbiota composition was analyzed by denaturing gradient gel electrophoresis for 16S rRNA gene followed by principal component analysis. Furthermore, immune functions and cytokine concentrations were analyzed using flow cytometry, ELISA or multiplex bead assay. We found that a single oral inoculation of GF mice at three weeks of age permanently changed the gut microbiota composition, which was not possible to achieve at one week of age. Interestingly, the ex-GF mice inoculated at three weeks of age were also the only mice with an increased pro-inflammatory immune response. In contrast, the composition of the gut microbiota of ex-GF mice that were co-housed with SPF mice at different time points was similar to the gut microbiota in the barrier maintained SPF mice. The existence of a short GF postnatal period permanently changed levels of systemic regulatory T cells, NK and NKT cells, and cytokine production. In conclusion, a time window exists that enables the artificial colonization of GF mice by a single oral dose of caecal content, which may modify the future immune phenotype of the host. Moreover, delayed microbial colonization of the gut causes permanent changes in the immune system.

## Introduction

The intestinal microbiome regulates many aspects of host biology, including development of the immune system [1], [2]. Gut microbiota heterogeneity, especially the presence and/or absence of certain strains of bacteria, is therefore an important factor contributing to phenotypic variation in animal models of human diseases [3], [4]. In a recent study, Ivanov et al. [5] showed that mice from Taconic Farms had a higher frequency of Th17 cells compared with mice from Jackson Laboratory, due to the presence of uncultivable segmented filamentous bacteria (SFB) in the gut of mice from Taconic. Mono-colonization of germ-free (GF) mice with SFB induced production of several pro-inflammatory cytokines in the gut mucosa compared to either GF mice or to mice colonized only with cultivable microbial species [6]. Interestingly, addition of this particular bacterium to a specific pathogen-free (SPF) bacterial cocktail used for mice colonization significantly increased their sensitivity to intestinal inflammation in a transfer model of colitis as compared to either use of the cocktail alone or to mono-colonization with SFB [7]. Furthermore, Kriegel and colleagues [8] demonstrated that diabetes incidence, but not insulitis score, was lower in SFB-positive females of non obese diabetic (NOD) mice compared to an incidence of 91% in SFB-negative NODs. By modifying the gut microbiota composition in rodent models, it may, thus, become possible to manipulate host immunity in order to promote or prevent certain disease traits. Accordingly, in diabetes-prone Bio-breeding rats it was possible to modify diabetes incidence in association with an altered gut cytokine profile by feeding them strong diabetes-promoting antigens through diet or lipopolysaccharide (LPS) in the first week of life [9]. However, studies addressing the importance of the initial intestinal colonizers on future immune phenotype are rather rare.

It is reasonable to assume that either GF or altered microbial conditions within the first weeks of life disrupt the early development of immune functions, while later in life the immunity remains stable with far less susceptibility to change by microbial influence [10]. This idea is supported by the hygiene hypothesis [11], [12], which postulates that microbial exposure in early postnatal life is important for the proper development of immuno-regulatory mechanisms and, thus, prevent inappropriate T cell responses and inflammatory diseases later in life.

The aim of this study was to investigate how different patterns of artificial colonization influence development of the immune system. For this purpose, we colonized GF pups at different time points with SPF microbiota from a different animal facility, and analyzed the gut microbiota composition and immune response later in life.

## Results

### Time of colonization influences gut microbiota composition

**Figure 1:**
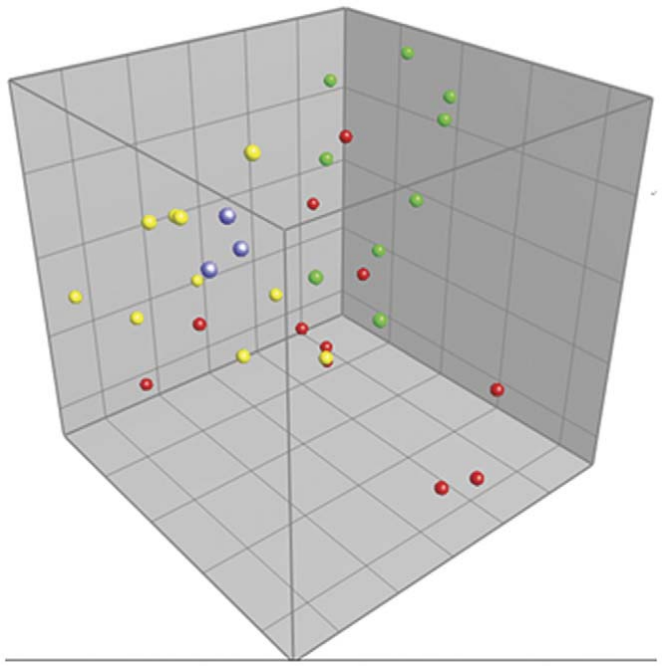
Gut microbiota comparison of inoculated mice. Principal Component Analysis (PCA) plot based on DGGE profiles of 16S rRNA gene PCR derived amplicons of feces samples collected from mice in the inoculation experiment at nine weeks of age. Germ-free (GF) mice were inoculated at one week of age (red balls) or at three weeks of age (yellow balls) with a bacteria suspension made from caecal content of specific pathogen-free (SPF) mice from Taconic Farms (blue balls illustrate different aliquots of the suspension). Their gut microbiota was compared with SPF mice raised in our barrier (green balls). ANOVA based on Principal Component (PC) 1 explaining 14.7% of the variance confirmed a significant difference between mice inoculated at three weeks of age and all other mice (*p*<0.01). No significant differences of PC values were found between ex-GF mice inoculated at three weeks of age and the suspension aliquots used to inoculate these mice with. DGGE: Denaturing Gradient Gel Electrophoresis.

The gut microbiota composition was compared after inoculating GF pups with caecal contents from SPF mice from Taconic Farms at different time points or after co-housing GF pups with SPF mice raised in our animal facility. After denaturing gradient gel electrophoresis (DGGE) of feces samples, principal component analysis (PCA) revealed significant differences between mice inoculated at three weeks of age and mice raised in our animal facility (Fig. 1). Moreover, gut microbiota composition of these inoculated mice closely resembled the DGGE profiles of the suspension aliquots used for conventionalization, which differed from the resident microbiota found at our animal facility. In contrast, the gut microbiota of mice inoculated with the same suspension at one week of age was not different from mice raised in our animal facility.

**Figure 2:**
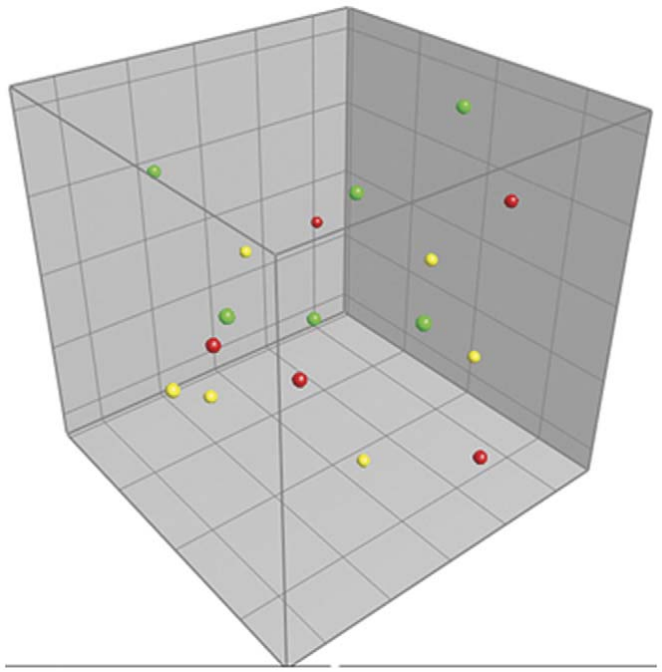
Gut microbiota comparison of co-housed mice. Principal Component Analysis (PCA) plot based on DGGE profiles of 16S rRNA gene PCR derived amplicons of feces samples collected from mice in the co-housing experiment at nine weeks of age. Germ-free (GF) mice were conventionalized at one week of age (red balls) or at three weeks of age (yellow balls) by co-housing them with specific pathogen-free (SPF) mice (green balls). No differences in gut microbiota composition were detected between the SPF and co-housed ex-GF mice. DGGE: Denaturing Gradient Gel Electrophoresis.

To ensure that the altered microbiota in the mice inoculated at three weeks of age was caused by inoculating a different microbiota and not solely due to a delayed colonization of environmental bacteria, GF mice were conventionalized only by co-housing them with SPF mice raised in our animal facility without any microbiota inoculations. Intestinal microbiota of ex-GF mice that were co-housed with SPF mice did not show differences to barrier maintained SPF mice (Fig. 2). Thus, delayed colonization had no effect on the gut microbiota composition. Neither gender nor housing in different cages had an impact on the gut microbiota composition in either type of experiment (results not shown).

### Colonization of GF mice at three weeks of age with microbiota from Taconic mice results in pro-inflammatory tuning of the immune system

**Figure 3:**
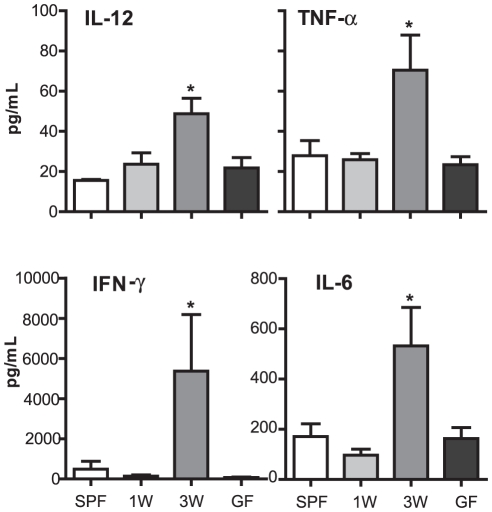
Microbial inoculation at three weeks of age results in pro-inflammatory tuning of the immune system. Cytokines were measured in mesenteric lymph node (MLN) cell culture supernatants and analyzed by use of FlowCytomix Multiplex Th1/Th2 10plex. MLN were isolated from specific pathogen-free (SPF) mice (SPF), germ-free (GF) mice (GF) and ex-GF mice inoculated with a caecal microbiota suspension at one (1W) or three weeks (3W) of age. The lymphocytes were stimulated for 24 hrs with 1 µg/mL lipopolysaccharide (LPS) from *Escherichia coli* O26:B6 before the supernatants were used for analysis. Only cytokines with a significant difference among groups are illustrated. Error bars represent the SEM. * represents *p*<0.05 compared with SPF mice.

Mesenteric lymph node (MLN) cells were isolated from GF, inoculated ex-GF, and SPF raised mice and stimulated using LPS. Cells from mice inoculated at three weeks produced significantly more IL-12, TNF-α, IFN-γ, and IL-6 compared to any other group (Fig. 3). Interestingly, this was the only group of mice which also had an altered gut microbiota. The other measured cytokines were below detection limit of the assay.

### An early postnatal germ-free period changes immune system regulation

**Figure 4:**
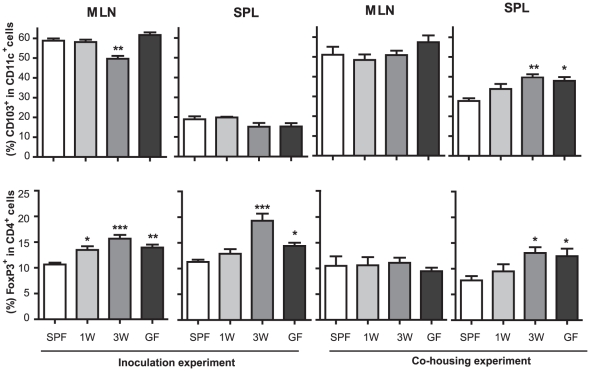
A germ-free postnatal period affects long-term immune-regulatory homeostasis. Percentages of regulatory T cells (CD4^+^FoxP3^+^) and tolerogenic dendritic cells (CD103^+^CD11c^+^) in cells isolated from spleen (SPL) and mesenteric lymph node (MLN) were determined by flow cytometry. Specific Pathogen Free mice (SPF), germ-free mice (GF) and ex-GF mice inoculated with a caecal microbiota suspension or co-housed with SPF mice at one (1W) or three weeks (3W) of age are illustrated. Error bars represent the SEM. * represents *p*<0.05, ** <0.01, *** <0.001 compared with SPF mice.

To analyze the influence of different colonization patterns, we analyzed the numbers of tolerogenic DCs (CD11c^+^CD103^+^) and regulatory T cells (Treg; CD4^+^FoxP3^+^) in both spleen and MLNs. We found that while tolerogenic DCs are not much changed in the inoculation experiment, GF mice and ex-GF mice in the co-housing experiment have significantly higher proportions of these cells in the spleen (Fig. 4). We observed a similar trend for Tregs in MLNs of ex-GF inoculated mice and in the spleen of both inoculated and co-housed mice (Fig. 4). These results show that means of colonization (single gavage versus co-housing) have no major impact on systemic tolerance induction, and that an early GF period permanently changes its development.

**Figure 5:**
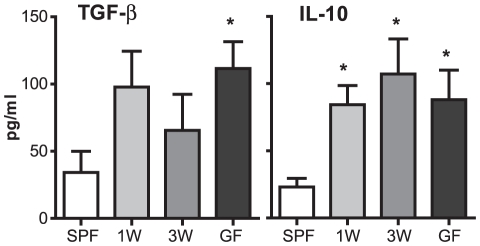
A germ-free postnatal period increases serum regulatory cytokines. Analyses of serum cytokines were performed by ELISA. Serum was extracted from Specific Pathogen Free (SPF) mice, germ-free (GF) mice and ex-GF mice co-housed at one (1W) or three weeks (3W) of age with SPF mice. Error bars represent the SEM. * represents *p*<0.05 compared with SPF mice.

Next, serum levels of TGF-β and IL-10 in the co-housing experiment were analyzed. In addition to higher proportions of Tregs in the spleen, higher serum levels of these regulatory cytokines were detected in GF and co-housed ex-GF mice compared to SPF raised mice (Fig. 5). These data confirmed the induction of regulatory immunity following a short postnatal GF period of only one or three weeks.

### An early postnatal period without microbiota alters the systemic mononuclear cell populations

**Figure 6:**
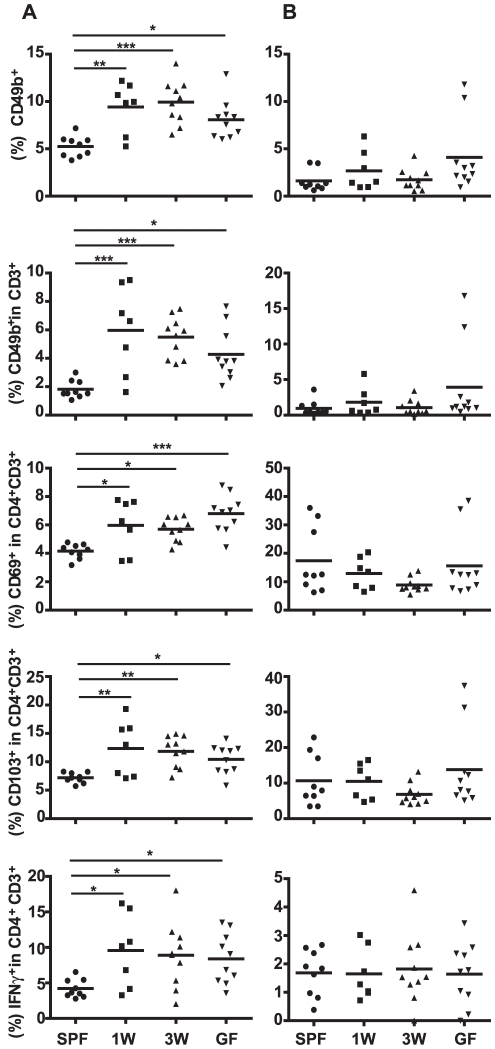
A germ-free postnatal period alters systemic mononuclear cell populations. Distribution of cell populations in spleen (A) and mesenteric lymph node (B) sampled from mice in the co-housing experiment were measured by flow cytometry. Germ-free (GF) mice were conventionalized at one week of age (1W) or at three weeks of age (3W) by co-housing them with Specific Pathogen Free mice (SPF) raised in our barrier. A group of mice remained germ-free (GF). Percentages of NK cells (CD3^−^CD49b^+^), NKT cells (CD3^+^CD49b^+^), CD69^+^ and CD103^+^ T cells (CD3^+^CD4^+^) and IFN-γ producing T cells (CD3^+^CD4^+^) are illustrated. Lymphocytes were cultivated for 4 hrs with 50 ng/mL PMA and 750 ng/mL Ionomycin (Sigma-Aldrich) in the presence of BD GolgiStop before cells were harvested and intracellular IFN-γ stained. The mean is illustrated. * represents *p*<0.05, ** <0.01, *** <0.001 compared with SPF mice. PMA: Phorbol 12-Myristate 13-Acetate.

Next, it was analyzed how timing of colonization influences development of the immune system. Mononuclear cells in the spleens isolated from GF, co-housed ex-GF, and SPF raised mice showed the same distribution among the groups as the serum cytokines. Higher relative amounts of NK cells (CD3^−^CD49b^+^), NKT cells (CD3^+^CD49b^+^), CD4 T cells with regulatory markers CD69 or CD103, and IFN-γ-producing CD4 T cells were detected in spleens, but not in MLNs, of both GF and co-housed ex-GF mice compared to SPF mice (Fig. 6). Therefore, the absence of proper host-microbe interaction during the first weeks of life is enough to permanently change the immune profiles of mice.

## Discussion

A large amount of literature generated in humans and gnotobiotic rodents have demonstrated that the type of microbial exposure can influence the immune system [13]. It is increasingly acknowledged that manipulating with the colonization process, e.g. by administration of probiotics to newborns or preterm infants, may improve health of the host in infancy and adulthood [14], [15]. However, our understanding of how timing of this colonization alters the ability of microbes to colonize and immune system reactivity later in life is still incomplete [16].

In the present study, we used caecal content of mice obtained from Taconic's barrier unit (different from the microbiota in our barrier facility) to permanently change the microbiota composition. This was achieved by single oral inoculation of GF mice at three weeks of age. Thus, the resulting microbiota composition residing in the gut corresponded to the one in the inoculum rather than the one in our barrier animal facility. Protected housing in isolators was no longer needed after inoculation to keep the gut microbiota stable, which is in agreement with previous findings showing that the established microbiota in the adult gut is less susceptible to change by environmental stimuli [17], [18]. Interestingly, similar inoculation at one week of age did not changed the adult microbiota composition, which turned out to be similar to the one found in our facility. The striking difference in colonization success between one- and three weeks old mice could be explained by changes in gut physiology during this time. These changes include increase in gut redox potential, changes in peristalsis and secretions, mucosal immune response (i.e. expression of certain pattern recognition receptors), and diet, which all control the colonization pattern of the immature intestine [19]. The differences in diet between one- and three-weeks old mice are of particular interest, because weaning has striking impact on gut microbiota [20]. The lack of colonization success at first week of age could be mediated by modulators of the gut microbiota composition, such as glycans (complex carbohydrates) and antimicrobial factors, known to be found in the maternal milk [21]. These factors probably inhibited the establishment of the one-time inoculated bacteria in the one week old mouse gut, letting the mice to be colonized by environmental microbes instead. Furthermore, we found that delayed colonization did not alter the microbiota's ability to colonize, because the ex-GF mice co-housed with SPF mice had the same gut microbiota as the SPF mice raised in the barrier. Thus, the altered gut microbiota in the ex-GF mice inoculated at three weeks of age was not just due to a delayed colonization, but because of a successful colonization of the inoculation suspension.

Next, we analyzed how these different colonization patterns change the immune system reactivity. The ex-GF mice inoculated at three weeks of age generated a pro-inflammatory biased immune system; most likely, directed by their altered gut microbiota composition. This has also been observed previously in Taconic SPF mice together with high levels of Tregs [22], but in the present study the high Treg levels were also observed in the GF mice, which indicate that this was a consequence of the late colonization rather than its modified microbiota. This seems reasonable as the infant gut is immature and in a state favoring the development of regulatory mechanisms in response to environmental stimuli [23], [24], which also makes the postnatal period an excellent time point to modify commensal-regulating pathways generating life-long alterations in the immune system.

In 1989, Strachan suggested that decreased microbial burden caused by improvement of hygienic standards in developed countries can lead to a higher incidence of immune-mediated diseases [12]. It is noteworthy that even though the gut microbiota in the co-housed ex-GF mice was not influenced by the early GF period, alterations in the systemic immune system still occurred. Higher serum levels of regulatory cytokines and higher relative amounts of several mononuclear cell populations were detected in spleens of GF and co-housed ex-GF mice compared to SPF raised mice. Even though the differences were subtle, they all showed the same pattern in the systemic immune populations. This shows that acquisition of microbial components in the gut immediately after birth plays an important role in directing future host immune profile far beyond the gut, which is also supported by the previous finding of a microbial signaling effect on systemic immunity [25]. Lack of appropriate microbial stimuli in the postnatal period may, thus, permanently alter important immune functions. Interestingly, a decrease in bacterial burden in early life by use of broad spectrum antibiotics also significantly decreases the expression of gut microbial sensors, such as Toll-like receptor 2, 4, and 5 [26]. This may explain why infants given antibiotics early in life seem to have an increased risk of immune-mediated disease later in life [27]. Further studies are needed to elucidate how the pattern of early microbial exposure influences the development of inflammatory and autoimmune diseases.

In conclusion, there is a time window at about three weeks of age, which enables the artificial colonization of GF mice by a single oral dose of caecal content. In constrast, colonizing mice by co-housing them following a short GF period does not alter the gut microbiota composition. Nevertheless, delayed colonization of either type causes permanent changes in immune system reactivity, which may downgrade the results of experiments performed on first generation of colonized animals. More research is needed to complete our understanding of how different components in a complex microbial community shape future host immune functions during critical windows of intestinal maturation.

## Materials and Methods

The experiment was carried out in accordance with the Council of Europe Convention European Treaty Series (ETS) 123 on the Protection of Vertebrate Animals used for Experimental and Other Scientific Purposes, and the Danish Animal Experimentation Act (LBK 1306 from 23/11/2007). The study was approved by the Animal Experiments Inspectorate, Ministry of Justice, Denmark (License number: 2007-561-1434 C3) as well as by the Laboratory Animal Care and Use Committee of the Institute of Microbiology, Academy of Sciences of the Czech Republic (Approval ID: 053/2010). All efforts were made to minimize the number of animals used and to minimize suffering.

### Animals

For the inoculation experiment GF outbred Swiss Webster (SW) mice (Taconic, NY, USA) were reared in sterilized plastic film isolators at the Faculty of Life Sciences, KU, Denmark. The mice had free access to an autoclaved Altromin 1314 diet (Altromin, Lage, Germany)) and water. The pups were kept GF for either one or three weeks after birth before they were inoculated with a bacterial suspension (described in the next paragraph) and subsequently housed in our barrier protected animal facility (Faculty of Life Sciences, University of Copenhagen, Denmark) under standard conditions in open cages without filter lids. The mice were compared with a GF control group and with SPF offspring from ex-GF SW mice conventionalized in our animal facility. The same diet continued throughout the entire experiment to all groups of mice.

In a second experiment GF BALB/c mice were reared in isolators under GF conditions at the Department of Immunology and Gnotobiology (Novy Hradek, Czech Republic) and fed *ad libitum* with Altromin 1414 diet (Altromin, Lage, Germany) sterilized by irradiation. These mice were not inoculated with any bacterial suspension, but they were conventionalized by co-housing with SPF BALB/c mice at the age of one or three weeks of age (co-housing experiment) and compared to GF and SPF BALB/c mice on the same diet. Establishment and control of GF breeding was as previously described [28]. Each group consisted of approximately ten mice with half male and females and all mice were euthanized at eight-nine weeks of age.

### Bacterial suspension and inoculation

Two male and two female SPF SW mice were euthanized immediately upon arrival from Taconic farms, and caecal contents were extracted and pooled into 50 mL PBS suspension. After thoroughly mixing and addition of DMSO (1∶200), the suspension was divided into homogeneous aliquots and frozen at −40°C until further use for inoculation. Twenty µL of the bacterial suspension was inoculated orally in one week and three weeks old GF SW mice using a 100 µL pipette with autoclaved tips. At least two litters per group were included in the experiment, and the mice were housed in one cage per litter together with their mother until weaning at four weeks of age. The mothers were not inoculated with the suspension and were killed when the pups were weaned and males and females separated into two cages per litter.

### Gut microbiota composition

Aliquots of the inoculation suspension and feces samples aseptically obtained from BALB/c and SW mice at the time of their euthanasia were analyzed by Denaturing Gradient Gel Electrophoresis (DGGE) as described previously [29]. Briefly, cellular DNA extracted with QIAamp DNA Stool Mini Kit (Qiagen, Hilden, Germany) according to manufacturer's instructions, was used as template for Polymerase Chain Reaction (PCR), using primers (PRBA338fGC and PRUN518r) specific to the V3 region of the 16S rRNA gene. Amplicons were separated by DGGE using a 9% poly-acrylamide gel containing a 30%–65% linear chemical gradient (where 100% correspond to 7M urea and 40% formamide in milliQ water). After 16 hours of electrophoresis the gels were stained with SYBR gold and photographed. Finally, the resulting DGGE profiles were analyzed using BioNumerics Version 4.5 (Applied Maths, Sint-Martens-Latem, Belgium).

### LPS stimulation of MLN lymphocytes

Isolated MLN cells from SW mice in the inoculation experiment were cultivated in 96 well plates in culture medium at a concentration of 1×10^6^ cells/mL. LPS from *Escherichia coli* O26:B6 (Sigma-Aldrich, St. Louis, MO) was added to a final concentration of 1 µg/mL. The plate was placed in a humidified 5% CO_2_ incubator at 37°C for 24 hours before the supernatants were harvested for cytokine analysis.

### Determination of cytokine production by ELISA and multiplex bead assay

Cytokine analyses on MLN culture supernatants were performed using the mouse Th1/Th2 10plex FlowCytomix Multiplex (eBiosciences, San Diego, CA). Beads for IFN-γ, IL-1α, IL-2, IL-4, IL-5, IL-6, IL-10, IL-17, GM-CSF and TNF-α were included in the kit and the procedures were performed according to manufacturer's instructions. Samples were analyzed using Accuri C6 flow cytometer. Cytokine concentrations were calculated according to standard curves prepared in supplied software FlowCytomix Pro 2.4.

Commercial ELISA kits were used to evaluate changes in serum TGF-β (Invitrogen, Carlsbad, CA) and IL-10 (eBioscience) concentrations in mice in the co-housing experiment according to manufacturer's instructions.

### Cell Isolation and Flow Cytometry

Cells were isolated from spleen and MLN by aseptically squeezing the fresh organs in PBS between two microscope slides and subsequently passing the suspension through a 70 µm cell strainer. All single cell suspensions were stored on ice at all time. Spleen cells were resuspended in red blood cell lysis (ACK) buffer (0.15 M NH_4_Cl, 10 mM KHCO_3_, 1 mM EDTA monosodium pH 7.3) and incubated for 6 minutes. Then, cells were washed in sterile PBS and resuspended in culture medium (RPMI 1640 supplemented with 10% fetal calf serum (FCS), 2 mmol/L L-glutamine and 2 mmol/L penicillin and streptomycin; all from Sigma-Aldrich).

Surface staining of dendritic cells was performed for 30 min with the appropriate antibodies. For Treg staining, cells were first surface stained with anti-mouse CD4 antibody, then fixed, permeabilized and intracellular Foxp3 stained according to the manufacturer's protocol (eBiosciences). For intracellular cytokine staining cells isolated from BALB/c mice were incubated for four hours with 50 ng/mL Phorbol 12-Myristate 13-Acetate (PMA; Sigma-Aldrich) and 750 ng/mL Ionomycin (Sigma-Aldrich) in the presence of GolgiStop (0.7 µL/mL; BD Biosciences, San Jose, CA) in a humidified incubator at 37°C and 5% CO_2_. Cells were stained and fixed using the same protocol as described for Tregs. Antibodies were purchased from either eBiosciences or BD Biosciences and dilutions were optimized for all analyses. Analysis was performed using an Accuri C6 flow cytometer (Accuri Cytometers Inc, Ann Arbor, MI) and LSRII (BD Biosciences).

### Statistical analysis

GraphPad Prism version 5.02 (GraphPad Software, San Diego, CA, USA) was used for statistical analysis and *P*-values less than 0.05 were considered significant. The experimental groups were compared by either one-way ANOVA test with Tukey-Kramer post test or by Kruskal-Wallis test with Dunn's post test on data that did not assume a Gaussian distribution. DGGE profile comparison was performed by the Dice similarity coefficient with a band position tolerance and optimization of 1% using the Unweighted Pair Group Method with arithmetic Averages clustering algorithm (UPGMA) and by Principal Component Analysis (PCA).

## References

[1] Cebra JJ (1999). Influences of microbiota on intestinal immune system development.. Am J Clin Nutr.

[2] Tlaskalova-Hogenova H, Stepankova R, Hudcovic T, Tuckova L, Cukrowska B (2004). Commensal bacteria (normal microflora), mucosal immunity and chronic inflammatory and autoimmune diseases.. Immunology Letters.

[3] Pozzilli P, Signore A, Williams AJK, Beales PE (1993). Nod Mouse Colonies Around the World - Recent Facts and Figures.. Immunology Today.

[4] Vijay-Kumar M, Aitken JD, Carvalho FA, Cullender TC, Mwangi S (2010). Metabolic syndrome and altered gut microbiota in mice lacking Toll-like receptor 5.. Science.

[5] Ivanov II, Atarashi K, Manel N, Brodie EL, Shima T (2009). Induction of intestinal Th17 cells by segmented filamentous bacteria.. Cell.

[6] Gaboriau-Routhiau V, Rakotobe S, Lecuyer E, Mulder I, Lan A (2009). The key role of segmented filamentous bacteria in the coordinated maturation of gut helper T cell responses.. Immunity.

[7] Stepankova R, Powrie F, Kofronova O, Kozakova H, Hudcovic T (2007). Segmented filamentous bacteria in a defined bacterial cocktail induce intestinal inflammation in SCID mice reconstituted with CD45RBhigh CD4+ T cells.. Inflamm Bowel Dis.

[8] Kriegel MA, Sefik E, Hill JA, Wu HJ, Benoist C (2011). Naturally transmitted segmented filamentous bacteria segregate with diabetes protection in nonobese diabetic mice.. Proc Natl Acad Sci U S A.

[9] Scott FW, Rowsell P, Wang GS, Burghardt K, Kolb H (2002). Oral exposure to diabetes-promoting food or immunomodulators in neonates alters gut cytokines and diabetes.. Diabetes.

[10] Min B, Thornton A, Caucheteux SM, Younes SA, Oh K (2007). Gut flora antigens are not important in the maintenance of regulatory T cell heterogeneity and homeostasis.. Eur J Immunol.

[11] Musso G, Gambino R, Cassader M (2010). Obesity, diabetes, and gut microbiota: the hygiene hypothesis expanded?. Diabetes Care.

[12] Strachan DP (1989). Hay fever, hygiene, and household size.. BMJ.

[13] Reading NC, Kasper DL (2011). The starting lineup: key microbial players in intestinal immunity and homeostasis.. Front Microbiol.

[14] Mihatsch WA, Braegger CP, Decsi T, Kolacek S, Lanzinger H (2011). Critical systematic review of the level of evidence for routine use of probiotics for reduction of mortality and prevention of necrotizing enterocolitis and sepsis in preterm infants.. Clin Nutr.

[15] Lodinova-Zadnikova R, Cukrowska B, Tlaskalova-Hogenova H (2003). Oral administration of probiotic Escherichia coli after birth reduces frequency of allergies and repeated infections later in life (after 10 and 20 years).. Int Arch Allergy Immunol.

[16] Kaplan JL, Shi HN, Walker WA (2011). The role of microbes in developmental immunologic programming.. Pediatr Res.

[17] Friswell MK, Gika H, Stratford IJ, Theodoridis G, Telfer B (2010). Site and strain-specific variation in gut microbiota profiles and metabolism in experimental mice.. PLoS One.

[18] Palmer C, Bik EM, DiGiulio DB, Relman DA, Brown PO (2007). Development of the human infant intestinal microbiota.. Plos Biology.

[19] Mackie RI, Sghir A, Gaskins HR (1999). Developmental microbial ecology of the neonatal gastrointestinal tract.. Am J Clin Nutr.

[20] Lee A, Gemmell E (1972). Changes in the mouse intestinal microflora during weaning: role of volatile fatty acids.. Infect Immun.

[21] Newburg DS, Walker WA (2007). Protection of the neonate by the innate immune system of developing gut and of human milk.. Pediatr Res.

[22] Ivanov II, Frutos RL, Manel N, Yoshinaga K, Rifkin DB (2008). Specific microbiota direct the differentiation of IL-17-producing T-helper cells in the mucosa of the small intestine.. Cell Host Microbe.

[23] Mold JE, Michaelsson J, Burt TD, Muench MO, Beckerman KP (2008). Maternal alloantigens promote the development of tolerogenic fetal regulatory T cells in utero.. Science.

[24] Sudo N, Sawamura SA, Tanaka K, Aiba Y, Kubo C (1997). The requirement of intestinal bacterial flora for the development of an IgE production system fully susceptible to oral tolerance induction.. Journal of Immunology.

[25] Clarke TB, Davis KM, Lysenko ES, Zhou AY, Yu Y (2010). Recognition of peptidoglycan from the microbiota by Nod1 enhances systemic innate immunity.. Nat Med.

[26] Dimmitt RA, Staley EM, Chuang G, Tanner SM, Soltau TD (2010). Role of postnatal acquisition of the intestinal microbiome in the early development of immune function.. J Pediatr Gastroenterol Nutr.

[27] Marra F, Lynd L, Coombes M, Richardson K, Legal M (2006). Does antibiotic exposure during infancy lead to development of asthma?: a systematic review and metaanalysis.. Chest.

[28] Hrncir T, Stepankova R, Kozakova H, Hudcovic T, Tlaskalova-Hogenova H (2008). Gut microbiota and lipopolysaccharide content of the diet influence development of regulatory T cells: studies in germ-free mice.. BMC Immunol.

[29] Hufeldt MR, Nielsen DS, Vogensen FK, Midtvedt T, Hansen AK (2010). Variation in the gut microbiota of laboratory mice is related to both genetic and environmental factors.. Comp Med.

